# Current State of DNA Barcoding of Sciaroidea (Diptera)—Highlighting the Need to Build the Reference Library

**DOI:** 10.3390/insects13020147

**Published:** 2022-01-29

**Authors:** Jostein Kjærandsen

**Affiliations:** The Arctic University Museum of Norway, UiT—The Arctic University of Norway, P.O. Box 6050 Langnes, NO-9037 Tromsø, Norway; jostein.kjarandsen@uit.no

**Keywords:** DNA barcoding, Sciaroidea, reference library, integrative taxonomy, Nordic

## Abstract

**Simple Summary:**

DNA barcoding is a method by which a specific region of the mitochondrial genome is used to quantify genetic distances within and between animal species. Most DNA barcodes of the world are assembled on the Barcode of Life online database BoldSystems (BOLD). There, machine-generated barcode index numbers (BINs) are automatically assigned to clusters of specimens thought to represent species. I review the current state of DNA barcoding of the superfamily Sciaroidea, a diverse insect group consisting of close to 16,000 described fly species in eight families. To date, over 1.2 million specimens of Sciaroidea have been barcoded and the 56,648 assigned BINs on BOLD already represent 3.5 times the number of described species. Still, 95% of the BINs have currently no associated scientific name and very little effort has been put into building a quality-checked reference library where named species are linked to the BINs on BOLD. In the Nordic region, however, substantial progress is made towards building a complete reference library. While DNA barcoding has tremendous potential for advancing the knowledge for many diverse groups of insects, its potential will never be fully reached absent more engagement of trained taxonomists to build voucher collections, curate the reference libraries, and describe new species.

**Abstract:**

DNA barcoding has tremendous potential for advancing species knowledge for many diverse groups of insects, potentially paving way for machine identification and semi-automated monitoring of whole insect faunas. Here, I review the current state of DNA barcoding of the superfamily Sciaroidea (Diptera), a diverse group consisting of eight understudied fly families where the described species in the world makes up some 10% (≈16,000 species) of all Diptera. World data of Sciaroidea were extracted from the Barcode of Life online database BoldSystems (BOLD) and contrasted with results and experiences from a Nordic project to build the reference library. Well over 1.2 million (1,224,877) Sciaroidea specimens have been submitted for barcoding, giving barcode-compliant sequences resulting in 56,648 so-called barcode index numbers (BINs, machine-generated proxies for species). Although the BINs on BOLD already represent 3.5 times the number of described species, merely some 2850 named species (described or interim names, 5% of the BINs) currently have been assigned a BIN. The other 95% remain as dark taxa figuring in many frontier publications as statistics representing proxies for species diversity within a family. In the Nordic region, however, substantial progress has been made towards building a complete reference library, currently making up 55% of all named Sciaroidea BINs on BOLD. Another major source (31%) of named Sciaroidea BINs on BOLD comes from COI sequences mined from GenBank, generated through phylogenetic and integrative studies outside of BOLD. Building a quality reference library for understudied insects such as Sciaroidea requires heavy investment, both pre sequence and post sequence, by trained taxonomists to build and curate voucher collections, to continually improve the quality of the data and describe new species. Only when the BINs are properly calibrated by a rigorously quality-checked reference library can the great potential of both classical taxonomic barcoding, metabarcoding, and eDNA ecology be realized.

## 1. Introduction

**Figure 1:**
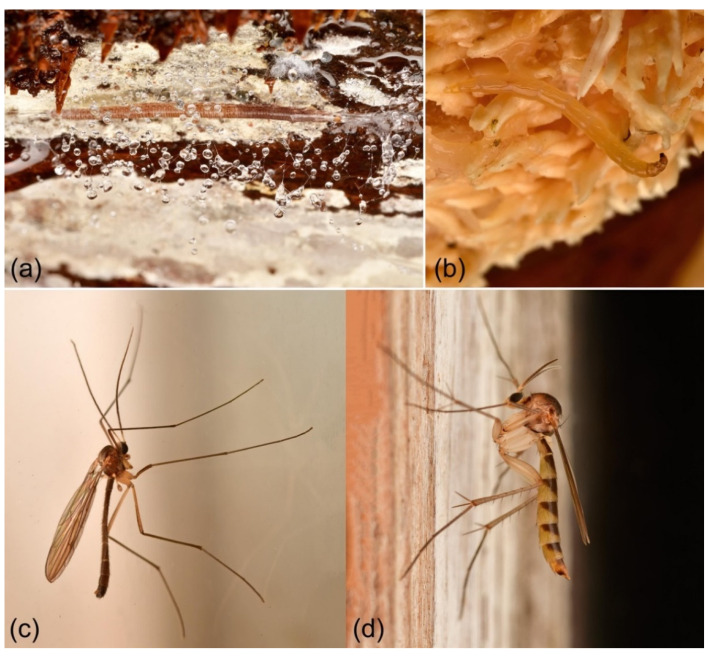
Examples of Sciaroidea flies. (**a**) Larva of cf. *Rocetelion humerale* (Zetterstedt, 1850), family Keroplatidae, spinning net under a huge decaying log of beech; (**b**) larva of *Sciophila varia* (Winnertz, 1864), family Mycetophilidae, spinning net under a sporophore of the mushroom *Hydnum repandum*; (**c**) adult male of *Bolitophila cinerea* Meigen, 1818, family Bolitophilidae; (**d**) adult female of *Allodiopsis rustica* (Edwards, 1941), family Mycetophilidae. All photos were taken by the author on October 2021 at Førde in Sveio municipality, Norway.

The superfamily Sciaroidea is a species-rich assemblage of lower flies belonging to the infraorder Bibionomorpha [1] that primarily are fungivorous at the larval stage (Figure 1), although many also are herbivorous (chiefly subfamily Cecidomyiinae) and some are predators. The superfamily currently makes up approximately 10% of all 160,000 described dipteran flies [2]. A large proportion, probably the vast majority, of the Sciaroidea fauna is still unknown to science, not at least within the superrich and little-studied family Cecidomyiidae, for which estimates based on DNA barcodes from Canada alone have postulated 16,000 species extrapolated up to 1.8 million species worldwide [3]. While that conjecture likely is a gross overestimate, it is no doubt that the Sciaroidea is a very successful evolutionary group, especially in amphitropic latitudes [4], that likely makes up considerably more species diversity than are presently described.

The taxonomic exploration of Sciaroidea flies dates all the way back to Carl Linné in Europe [4], and in the last 200 years, new species have been steadily discovered and described by taxonomists. While the earliest descriptions were superficial and largely rested on coloration patterns and wing venation, the deposition of type materials in museum collections made it possible for the next generation of taxonomists to re-examine and review them. Through the invention of better microscopes towards the end of the 19th century, it was discovered that their male terminalia revealed a minute but highly diverse world of stable characters, shown to be species specific for the great majority of the species. Females display less degree of visible morphological differentiation than males and are still often left uncovered. With this, their exploration entered a new phase where published illustrations accompanied with detailed descriptions could largely replace re-examination of type materials for identification.

**Figure 2:**
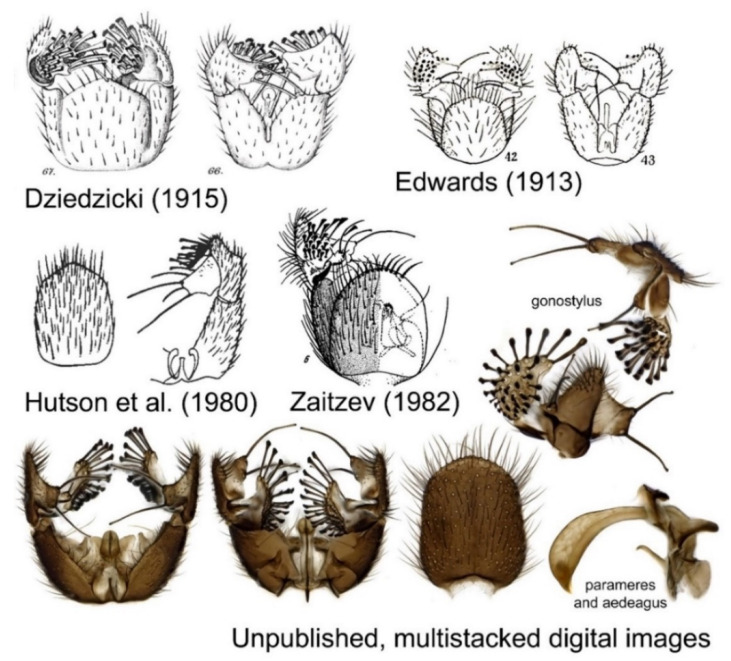
Example of historic illustrations over the last century for illustrating species-specific characters of the male terminalia for the fungus gnat species *Sciophila hirta* (Meigen 1818). The species’ terminalia has been illustrated by Edwards, Dziedzicki, Hutson et al. and Zaitzev [7,8,9,10]. Today, stacked digital images replace illustrations where more details and angles of their complex three-dimensional structures can be depicted with less work. By aid of DNA barcoding, this species is now about to be split into a complex of several semi-cryptic species that can only be safely separated based on details in their internal organs such as parameres and aedeagus, as such largely invalidating usage of previously published illustrations.

This phase has remained essentially unchanged for the last 100 years (Figure 2) until DNA barcoding based on molecular data entered the scene around the year 2000. Henryk Daniel Robert Dołęga Dziedzicki (1847–1921) at the Polish Academy of Science in Warsaw was among the very first to illustrate both the male and the female terminalia for each species in rigorous taxonomic revisions of genera of fungus gnats [5,6]. In the field of taxonomy, such revisionary works, as initiated by Dziedzicki, are of paramount importance that advanced the field and have been widely used and cited by taxonomists over a century after being published. Nowadays, such revisions sadly are notoriously scarce and for these kinds of thorough studies with a century-long impact, it is almost impossible to obtain funding in the modern academic environment with its ill-founded focus on short-term impact statistics. This has resulted in ever more scattered taxonomic literature with single or a few species described in each paper, making it quite hard for a new generation of taxonomists to learn the group and continue the taxonomic exploration.

With the development of DNA barcoding [11], an entirely new opportunity for taxonomic species exploration was born. Instead of year-long literature and morphological studies, the species can potentially be quantified and identified through machine-generated genetic sequencing. As it happens with most technological advances and quantification opportunities, DNA barcoding was quickly integrated with traditional taxonomy resulting in a boosted phase of new frontier exploration of species diversity worldwide and the detection of so-called cryptic species that are genetically distinct but cannot be identified by means of morphology.

**Figure 3:**
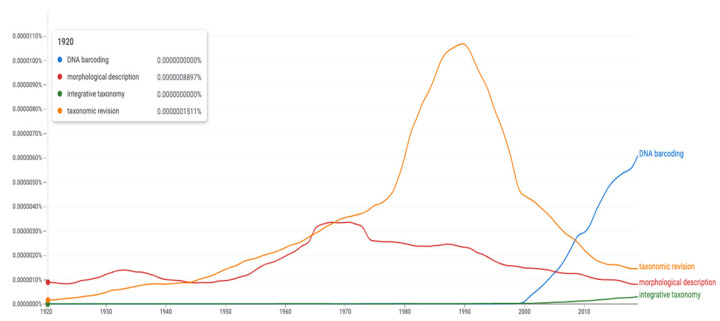
Google Ngram Viewer plot for the use of the terms “morphological description”, “taxonomic revision”, “DNA barcoding” and “integrative taxonomy” over the last century (1920 to 2019). Note that “morphological description” was more used hundred years ago compared to present day. Settings are “English 2019” dataset, “not case sensitive” and “smoothing 4” on a scale from 0 to 6. Link [13].

The dramatic change can be illustrated through a search on central taxonomic terms by use of Google Books Ngram viewer [12] (Figure 3); specifically, how the four terms “morphological description”, “taxonomic revision”, “DNA barcoding”, and “integrative taxonomy” have been used in the last 100 years (1920–2019 data). Although biased by the term, “morphological description” and perhaps even “taxonomic revision”, being partly used also outside the field of biology, the diagram makes a lot of sense. Usage of the term “morphological description” has been fairly stable with a broad peak in the mid-1960s to the early 1970s; thereafter, followed by a steady decline to the present level which is even lower than that of 100 years ago. Usage of the term “taxonomic revision” had a steady increase to high levels peaking around 1990 before a dramatic and fast decrease back to 1950s levels at present. The term “DNA barcoding” arose around the year 2000 and displays a steep, steady increase to present levels that are 4–20 times higher than those of the other terms used today. The term “integrative taxonomy” lags behind and is still today the least-used term of the four. The inflection point where the use of “DNA barcoding” exceeded that of “morphological description” occurred in 2005, while the inflection point where the use of “DNA barcoding” exceeded that of “taxonomic revision” occurred in 2008. This trend will probably continue although it is likely that we will see a leveling off through increased use of “integrative taxonomy”.

DNA barcoding has tremendous potential for advancing species knowledge and quantifying species-specific distributional and ecological properties for many diverse groups of insects [14,15], potentially paving the way for machine identification and semi-automated monitoring of whole insect faunas [16,17]. Imbedded in the original development of DNA barcoding was, however, an essential subgoal to build a reference library where quality-checked, named specimens identified by means of classical morphological methods and deposited in voucher collections are linked to their barcodes and so-called barcode index numbers (BINs) [18]. So far, rather modest funding and efforts have been allocated to this endeavor while more and more studies uncritically use BINs to represent proxies for species [19] or uncritically extract names from the Barcode of Life online database BoldSystems (BOLD) and GenBank without validating their sources and quality [20,21]. For instance, Svenningsen et al. [20], in a study detecting flying insects using car nets and DNA metabarcoding, claimed they documented 319 species not previously known from Denmark. When checking the species of Mycetophilidae on their list, it was found that all five species claimed new to Denmark were not new but appeared new due to misspellings, synonyms, and different genus combinations. An assessment of the taxonomic reliability of DNA barcodes in publicly available databases [22] provides compelling evidence of such data quality problems along with insufficient and unreliable annotation of taxonomic data.

Here, I will review the progress and status of DNA barcoding for the superfamily Sciaroidea with emphasis on the need to build a reference library and draw on experiences with building a reference library for Nordic fauna. While DNA barcoding and the BIN system on BOLD clearly is an efficient way of identifying genetic operational taxonomic units (gOTUs) of Sciaroidea (see Hartop et al. [23] for a discussion of weaknesses and alternative methods), its relation to Linnean scientific names established through the morphologically based taxonomic tradition is far from unequivocally clear and unambiguous.

## 2. Results and Discussion

### 2.1. The Hype around Blind Barcode Scanning

DNA barcoding has shown to be a tremendously successful tool to identify potential Sciaroidea species through the automatic barcode index number (BIN) system implemented on BOLD [3,24]. Yet, a major proportion of the assigned Sciaroidea BINs on BOLD still have no morphologically identified voucher specimen in a reference library (Table 1). Data pulled familywise from the taxonomy browser of BOLD, on the extraction date of 14 October 2021, revealed that well over 1.2 million (1,224,877) specimens of Sciaroidea have been submitted for barcoding, with a sequence success rate of 99%, giving barcode-compliant sequences resulting in 56,648 BINs. The public data portal is slightly smaller and has reported 1,025,065 published records forming 56,643 BINs with sequences from 76 countries deposited in 69 institutions. Among all these specimen records, only 92,769 (9%) have any form of an associated species name. While the 56,648 Sciaroidea BINs potentially represent 3.5 times the number of species than those that are currently described worldwide, merely 2843 scientific species names (including all used interim names and accessible private data) are associated with them. That means, assuming for simplicity, that there, in theory, could be a one-to-one relation between Linnean taxa and BINs, that for every named species in one BIN there are some 19 BINs representing unnamed, dark taxa [25]; or put another way, that only some 5% of the barcoded species have been identified to a species level.

Major barcoding projects in Costa Rica [28], Canada [3], and South Africa [19], have barcoded vast numbers of Sciaroidea, but so far little effort has been put into identifying the species. In Costa Rica, 145,208 barcoded Sciaroidea specimens represent 9543 BINs but have merely 10 identified species names. This can be expressed as a specimen to BIN ratio of approximately 15 and a BIN to named species ratio of some 954.

In Canada, 329,843 Sciaroidea specimens are barcoded, resulting in 16,053 BINs. Of these, merely 465 species are named, giving a specimen to BIN ratio of 21 and a BIN to named species ratio of 35. It can be noted that in Canada some of the named species appear to come from barcode associations via the Nordic reference library rather than from local expert identification. The author further assisted with assigning genus names to nearly all the 32,000 barcoded specimens of the family Mycetophilidae from Canada by means of an examination of specimen photos following each BIN.

A recent example of postponing the work to associate names to barcodes and BINs was demonstrated with efforts to DNA barcode the insect fauna of Krüger National Park in South Africa [19]. Among 36,229 barcoded Sciaroidea specimens resulting in 2448 BINs, only a single one of the BINs was assigned to a named species.

In Germany, a country with a strong tradition in taxonomy, the situation seems much better [29,30] where the intention to build a reference library is explicitly stated and trained taxonomists are hired to identify barcoded specimens, at least post-sequence. Still, among 16,433 barcoded Sciaroidea specimens and 2065 BINs from Germany on BOLD, only 553 species are currently publicly named, giving a specimen to BIN ratio of 8 and BIN to named species ratio of 4. Using BINs from Germany, Chimeno et al. [31] estimated the unknown German fauna of Cecidomyiidae to be between 62% and 71% and that of Sciaridae to be between 48% and 74%.

The latter is reflected in the entire BOLD archive where the unnamed Sciaroidea taxa are strongly biased towards the megadiverse and less-studied families Cecidomyiidae and Sciaridae, while for Mycetophilidae, Keroplatidae, and the remaining smaller families, a considerably larger proportion of BINs have already been assigned to named species (Table 1). The question then becomes who will carry out the labor-intensive post-sequence work to associate the millions of barcodes and thousands of BINs to named and undescribed species of Sciaroidea, or any other taxa for that matter, when this essential part of the barcoding enterprise is rarely included in the original projects nor in their funding plan. It seems at present that this endeavor is largely left for the underfunded and scarce community of endangered taxonomists [32] to engage in post-sequence at will [33], which, of course, may also be taken as an opportunity to boost integrative taxonomic work given that funding is allocated for it. The work required to achieve high-quality reference libraries for species-rich and understudied taxa such as the Sciaroidea can be likened to family-level taxonomic revisions. Much more effort and funding should be allocated to this endeavor rather than continue to support further frontier but scientifically blind mass-barcoding projects of dark taxa. A new field of “integrative barcode revisions” may be established and encouraged to reach the original goal of quality-checked reference libraries.

### 2.2. Integrative Sciaroidea Taxonomy and Ecology

Over the last decade, an increasing number of studies of Sciaroidea have used the integrative method to combine DNA barcodes with morphological studies in descriptive and revisionary works. This started with the use of DNA barcodes to associate immature stages [34] and females [35] to morphologically identify males. Later, DNA barcodes were increasingly used as a tool to aid in the discrimination of species for the families Mycetophilidae [36,37,38,39,40,41,42,43,44,45,46], Sciaridae [47,48,49,50,51,52,53,54,55,56,57,58], Cecidomyiidae [59,60,61,62,63], Keroplatidae [41,64,65], Ditomyiidae [66], and for a few taxa with an uncertain and contentious family placement often referred to as the Sciaroidea *incertae sedis* group [67,68]. DNA barcodes have also been shown to be a very efficient tool to associate females with identified males [35,44,45,46,47,48,49,50,51,52,53,54,55,56,57,58,59,60,61,62,63,64,65,66]. Some studies even used the integrative method to study the ecology of Sciaroidea taxa [69,70].

While these studies surely have helped build a rigorous reference library on BOLD, the species covered so far only make up a small fraction of the entire database.

### 2.3. The Nordic Initiative to Build a Reference Library of Sciaroidea

In the Nordic region, major efforts are being undertaken by local taxonomists to submit morphologically identified specimens, specifically with the aim to build the reference library on BOLD for their local native fauna. These efforts came about through a tight co-operation between the international Barcode of Life (iBOL, Ontario, Canada) via the local node, the Norwegian Barcode of Life (NorBOL, Trondheim, Norway), and the Norwegian Biodiversity Information Centre (NBIC, Trondheim, Norway) in Norway [24,49], and through similar efforts involving the Finnish Barcode of Life (FinBOL, Oulu, Finland) in Finland [26]. These initiatives specifically invited and economically supported local taxonomic experts to build a reference library and, as a result of this, the reference library is approaching full Nordic coverage for several of the Sciaroidea families (Table 1).

Combining all accessible private data with publicly available data on Sciaroidea from the Nordic region produced a dataset on BOLD of 14,908 submitted Sciaroidea specimens assigned to 2191 BINs. The author’s contribution of this is made available with this paper in the public dataset DS-NORSC (Supplementary Materials). While these efforts merely make up 1.2% of all Sciaroidea on BOLD and the BINs only represent 3.9% of all BINs on BOLD, the 1557 morphological identified species from the Nordic region make up 55% of all named Sciaroidea on BOLD, with the highest scores for the families Bolitophilidae (97%), Mycetophilidae (74%) and Sciaridae (55%) while the mega-rich family Cecidomyiidae (14%) is still poorly covered.

The overall rate of BINs divided by named species for all Sciaroidea from the Nordic region is 1.4 while the family Mycetophilidae even has more named species than assigned BINs. The latter comes from the fact that numerous rare and uncovered species were attempted to be barcoded but failed to give sequences due to aged samples of poor quality. In order to estimate how much of the local Nordic fauna is currently covered in these reference libraries, various checklists or estimates of the magnitude of the Nordic species diversity can be used as benchmarks.

Roslin et al. [26] published a comprehensive reference library of DNA barcodes for the arthropods of Finland and released a new identification tool based on this resource. Disappointingly, the DS-FINPRO library released in this study only covers some 20% of the Nordic Sciaroidea BINs, thus making the identification tool quite incomplete as a source for the identification of Finnish Sciaroidea at this point. However, with upgrades of all the Nordic BINs, the identification tool would have a near-complete coverage of several of the families.

Compared with Sciaroidea species published from Norway [24,27] the Nordic reference library covers 1.7 times the published species, with the number of BINs divided by published species ranging between 1.0 (Ditomyiidae) and 4.2 (Sciaridae) for individual families. Compared with the Swedish estimate of Sciaroidea species [4], the Nordic BINs make up 81%, where the best-covered family is Sciaridae with 1.28 times of BINs compared to its species estimate. The reference library has high scores for the families Mycetophilidae (99%), Bolitophilidae (95%), and Keroplatidae (90%), while Cecidomyiidae (49%) again has the weakest coverage.

A somewhat lower coverage is revealed when estimates of the total Nordic diversity are assembled from different sources. The reference library then has high scores for the families Bolitophilidae (90%), Mycetophilidae (84%), Sciaridae (80%), and Keroplatidae (72%) while Cecidomyiidae (34%) again has the weakest coverage.

One of the tools implemented on BOLD, called “barcode gap analysis”, calculates the difference between inter- and intraspecific genetic distances within a group of organisms (for an early discussion of the concept of barcode gaps see Meyer and Paulay [71]). Such an analysis performed for the Nordic dataset of Sciaroidea revealed a mean distance to the nearest neighbor of 6.97% while the mean intra-specific distance was 0.81%. This indicates on average some 45 COI base-pairs being different between closely related species of Sciaroidea. The largest distance to the nearest neighbor was 24.79%, between two species belonging to the families Cecidomyiidae and Keroplatidae, respectively. It must be emphasized, however, that such a barcode gap analysis needs carefully curated species data and is very prone to variations and errors in the names used in the data set, such as minor spelling errors or different versions of a name (e.g., a prefix such as cf. is occasionally used). Any misidentification will further obscure the results of the analysis.

#### Integrative Disclosures and Discoveries

On the surface, building a reference library may seem like a simple, straightforward process. Taxonomic experts use their long-built expertise to identify specimens, preferably pre-sequence but in many cases even post-sequence. Then, once a correct taxon name has been assigned to a BIN on BOLD subsequently added specimens may in theory simply be assigned the same name post-sequence. However, building hypotheses of species delimitation is a complex task that involves associating morphologically delimited species with available names, existing descriptions, and illustrations, often of poor quality. The next, integrative step to intertwine morphological identifications with the BIN assignments, adds further complexity that invokes reevaluations of the pre-sequence identifications. On top of that, a certain rate of practical mistakes is inevitably involved in the manual tasks that morphological identifications represent. As such, the post-sequence discrepancies may be sourced in an array of errors from simple operational mistakes to too broad or too narrow morphological delimitation of the sequenced species. The BINs, on the other hand, can neither be trusted to represent species at face value, and must be judged back against the morphological evidence.

The widespread lack of reference materials and reporting of taxonomic identifications procedures has long posed a challenge for replicability within the entomological literature [72,73]. The magnitude of mistakes in taxonomic identifications based on morphology has seldom been subjected to investigation; however, see MacLeod et al. [74] and Culverhouse et al. [75] for an introduction to the topic. With DNA barcoding on BOLD, reference materials are secured in voucher collections and an independent tool, the BIN assignments, can be used to estimate the magnitude and cause of corrections carried out during the integrative process to check and refine species hypotheses.

**Figure 4:**
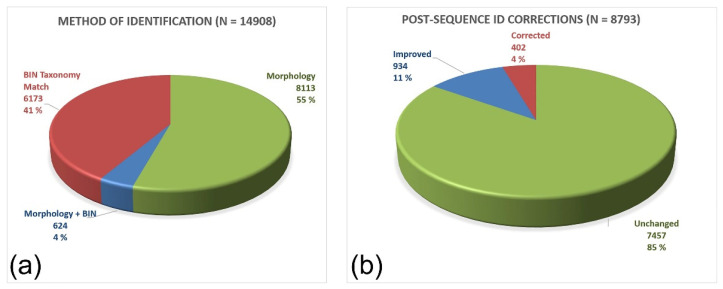
Method of identification (**a**) and post-sequence corrections (**b**) carried out on the Nordic dataset of Sciaroidea as of 15 September 2021. The entire Nordic dataset (*n* = 14,908) contains a large proportion of public data apparently identified only by BIN taxonomy matching. In our private data set (*n* = 8793), every submitted specimen was identified as much as possible pre-sequence and nearly every post sequence change in identification was noted on BOLD. Those sums to 402 (4.6%) corrections and 934 (10.6%) improved identifications while 7457 (84.8%) remains unchanged.

Among the dataset consisting of 14,908 Nordic barcodes of Sciaroidea, 8113 (55%) were identified by morphology by an expert pre-sequence, 6173 (41%) were identified through BIN or higher rank associations post-sequence, while 624 (4%) were identified by a combination of morphology and BIN associations (Figure 4a). A log kept on BOLD revealed that among 8793 barcoded specimens 85% kept their pre-sequence identifications unchanged while 934 (11%) had their identification precision improved and 402 (5%) were corrected post-sequence (Figure 4b). The correction rate is likely an underestimate since most of the changes were noted by the author, and other people involved did not necessarily note all their corrections. The error rate resulting from simple operational errors and mistakes in identifications is difficult to read out of the correction log, but it is not insignificant and represents something that both can and must be dealt with on BOLD. Similar to contaminations and lab mix-ups, these errors can in most cases be detected, flagged, and corrected, but this requires a thorough investment post-sequence involving iterative checking and comparison of ID trees and voucher specimens.

The 11% that had their identification precision improved largely concerns cases where unidentified larvae or females were submitted, but also many cases where the identification of males was uncertain and thus postponed to the post-sequence analysis. The species identification of larvae and females rests heavily on the identification of their corresponding males within the same BIN. In other words, the reference library is already during buildup used to associate specimens where these cannot be safely identified at the species level due to a lack of knowledge. Usually, such specimens were initially identified at the genus level, but for many larvae, only a family level identification was possible pre-sequence. It must be emphasized that the identification of these is only as good as the associated male identification, akin to those 41% submitted by other contributors that were assigned a name based only on BIN taxonomy matches.

Herein lies a great responsibility on those of us who assign Linnean species names to barcodes on BOLD. If the original identification is incorrect and remains unchecked and uncorrected for an extended period, such BIN taxonomy matches may serve to give it improper credibility which again reinforces the trust in the name and species hypothesis when a growing number of sequences are associated to a name. Again, heavy investments by trained taxonomists to perform the necessary quality checking is of paramount importance. With extended geographical coverage of a BIN, the complexity increases.

An example can be made from a very common species complex of the mycetophilid genus *Mycetophila* Meigen, 1803, widely distributed in the Holarctic region. The *Mycetophila fungorum* complex consists of at least 10 described species that are all quite difficult to distinguish from each other based on morphology [76,77]. On BOLD, an ID-clade representing these currently consists of 3735 specimens in seven different BINs. The author has identified a few Nordic specimens to the two common European species *Mycetophila fungorum* (De Geer, 1776) and *Mycetophila perpallida* Chandler, 1993. BIN taxonomy matching has likely, by large, extrapolated this to 3579 “identified” specimens where those identified to *Mycetophila fungorum* were later split into two BINs, one Holarctic and one Nearctic in distribution. While BOLD provides a list of identifiers, this largely contains names of BOLD staff unlikely to be able to identify these species as well as the BOLD ID Engine. In this case, at least 1604 identifications have likely ended up wrong such as *Mycetophila fungorum* in BIN BOLD:ACF2821, while most of those assigned to the true *Mycetophila fungorum* and to *Mycetophila perpallida* remain unchecked. A BOLD user can easily get the impression that the identification of thousands of specimens has been quality-checked for these species.

**Figure 5:**
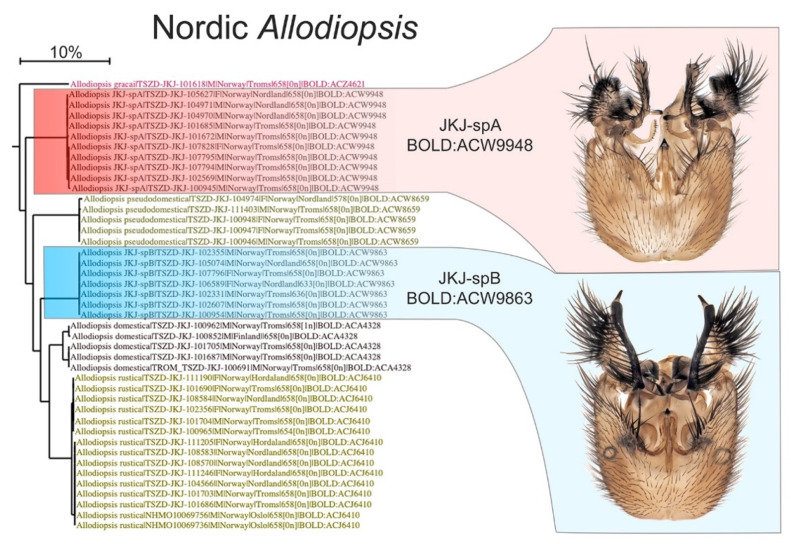
Example of perfect match between morphological identification, DNA barcodes, and BIN assignments on BOLD. Six species of the genus *Allodiopsis* Tuomikoski, 1966, family Mycetophilidae, showed little interspecific barcode variance and were assigned to BINs with distinct barcode gaps. Two of the species, identified *pre-sequence* as new to science, were confirmed by barcodes and BINs.

After checking for and correcting operational errors and mistakes in the *pre-sequence* identifications, the great majority of results from DNA barcoding came back with a near-perfect match between the morphological identification and their BIN assignment on BOLD. This is very encouraging. The Mycetophilid genus *Allodiopsis* Tuomikoski, 1966, can serve as an example of a perfect match (Figure 5) where four described species and two assumed new to science were identified pre-sequence. All these six species showed little interspecific variation and were each assigned to a single BIN with distinct barcode gaps.

However, the merging and mixing of BINs in relation to named species may be sourced in cases of introgression, incomplete lineage sorting, or if Linnean species are inappropriately assigned too many names [78]. In some cases, the morphological differentiation exceeded the COI differentiation, resulting in BIN sharing of distinctly different species. Within Sciaroidea, this was first acknowledged by Kurina et al. [36] who described two new species of the genus *Neuratelia* Rondani, 1856, where one of the new species shared barcodes with the type species for the genus. In the Nordic region, we have currently identified 18 such, double-checked and confirmed, cases of BIN sharing for Nordic species of the family Mycetophilidae, involving 15 different genera.

**Figure 6:**
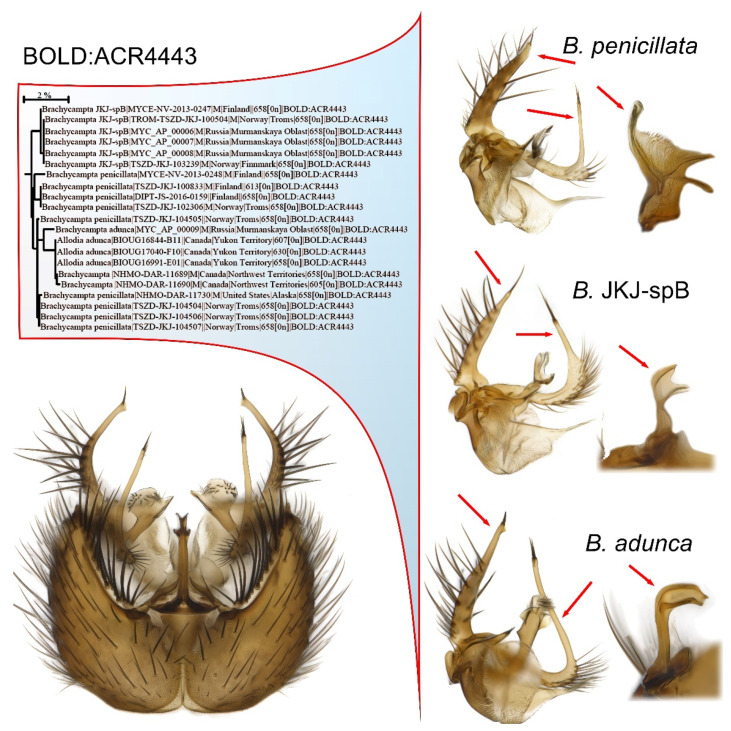
Example of BIN sharing of three morphologically distinct species. The *Brachycampta adunca*-complex merged two described species and one considered to be new to science into the same BIN, BOLD:ACR4443. Within the BIN the three morphotype species are only partly resolved for *Brachycampta* JKJ-spB while *Brachycampta adunca* Zaitzev, 1992 is nested within *Brachycampta penicillata* (Lundstrom, 1912). This complex belongs to the genus *Brachycampta* Winnertz, 1864, recently reinstated by Magnussen et al. [79], thus the *sensu lato* genus name *Allodia* Winnertz, 1864, is still used by other BOLD users. The images depict the male terminalia of *Brachycampta adunca* in ventral view and details of the gonostylus and hypandrial lobe for the three involved species to the right.

An example is the BIN BOLD:ACR4443 that embeds two described species and a third considered pre-sequence to be new to science (Figure 6). This complex belongs to the recently reinstated genus *Brachycampta*, Winnertz, 1864 [79], thus the *sensu lato* genus name *Allodia* Winnertz, 1864, is still used by other BOLD users. In this complex, the variation seen in the relative length and outline of the branches of the male gonostylus, as well as differences in the hypandial lobe, is way above the minimum level normally regarded as species-specific characters for the genus. A certain segregation into these three morphotypes is seen in the ID tree, but it is not fully resolved as *Brachycampta adunca* Zaitzev, 1992, is nested within *Brachycampta penicillata* (Lundstrom, 1912). *Brachycampta* JKJ-spB, however, could possibly be manually calibrated to a separate BIN cluster although the genetic distance to the other two is under 1%. When the BIN registry was introduced [18], such manual splitting of BINs by experts was actually suggested: “Where two or more species with diagnostic substitutions have been merged in a BIN, an expert may divide this BIN by specifying the position of the diagnostic nucleotides that allow their discrimination. These new divisions are treated as partitions of the existing BIN by extending the URI with a decimal value.” [18] (p. 11). To date, the author has not seen this option being implemented, but plans to suggest it for some of the Nordic cases of BIN sharing among Sciaroidea species.

An opposite situation, where one morphological species is split into several BINs, is much more frequently encountered. Such BIN splits initiate the hunt for new semi-cryptic species. Quite often, the BIN split is eventually confirmed with previously overlooked morphological differences. However, to clarify such cases requires thorough, time-consuming checking comparable to revisionary taxonomic work. Several specimens, preferably with a geographic spread, need to be examined in order to confirm if such minor morphological differentiations are consistent.

**Figure 7:**
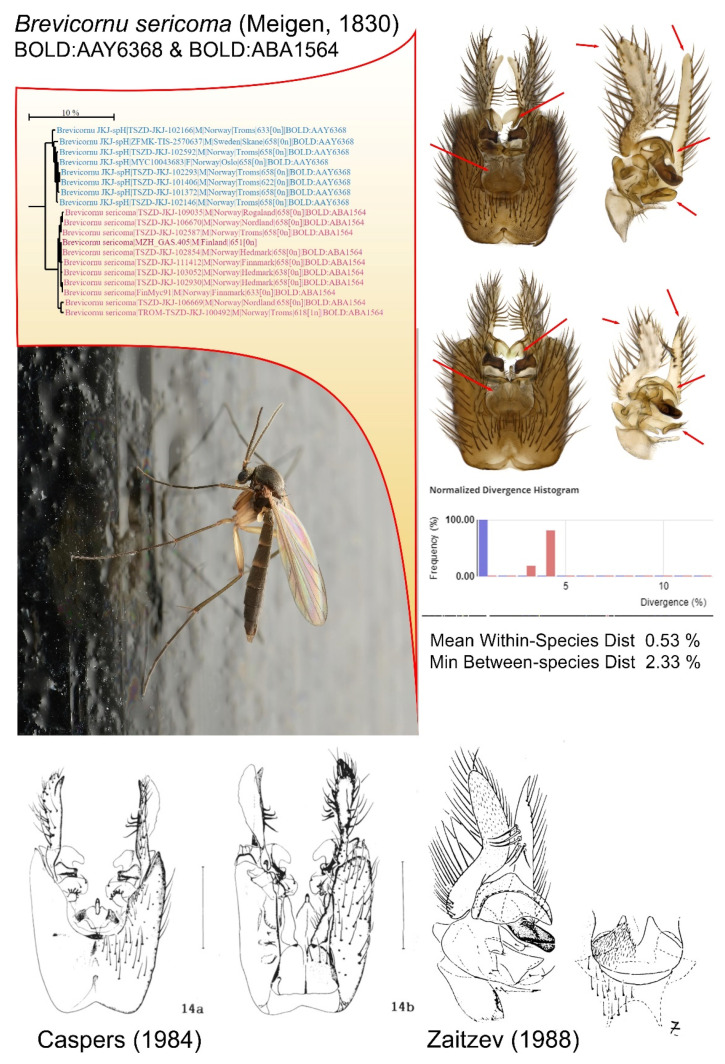
Example of BIN splitting of the Mycetophilid species *Brevicornu sericoma* (Meigen, 1830). What was previously regarded as one morphological species is split into two distinct BINs, although with a rather small between-species distance of 2.33% and a quite large mean within-species distance of 0.53%. Minor morphological differentiation is found between the BINs but also variation in some characters that do not follow the BIN segregation. Depicted at the bottom are illustrations published by Caspers [80] conforming to BIN BOLD:AAY6368 and Zaitzev [81] conforming to BIN BOLD:ABA1564.

An example of such a case is the mycetophilid species *Brevicornu sericoma* (Meigen, 1830). Right from the beginning, barcodes of this species were split into two different BINs. This sometimes happens when only a few sequences are present, and these BINs subsequently merge into one BIN when more sequences are added. However, in this case, adding new sequences only reinforced the split into the two BINs BOLD:AAY6368 and BOLD:ABA1564 (Figure 7), where the average within-species distance of 0.53% is quite large compared to the minimum between-species distance of 2.33%, but still distinct enough to split them into two BINs.

Examination of specimens from both BINs revealed some minute differences but also some apparent morphological variation within and among each BIN. For instance, the length of the dorsal branch of gonostylus appears to vary across and within the BINs. Studies of published illustrations revealed some differences in the inner sclerites of the gonostylus and in the hypandrial lobe and these appear to be consistent with the two BINs. This speciation case is still not concluded and even deciding which of the two BINs represent specimens of the originally described species is difficult since the type of material after Meigen is regarded as lost. A lesson learned from this exercise is that DNA barcoding may lead to new species discoveries, but in several cases, rather than giving clear answers, the uncertainties often still linger over many cases of shallow BIN splits where a parallel vagueness is seen in their morphological evolution.

**Figure 8:**
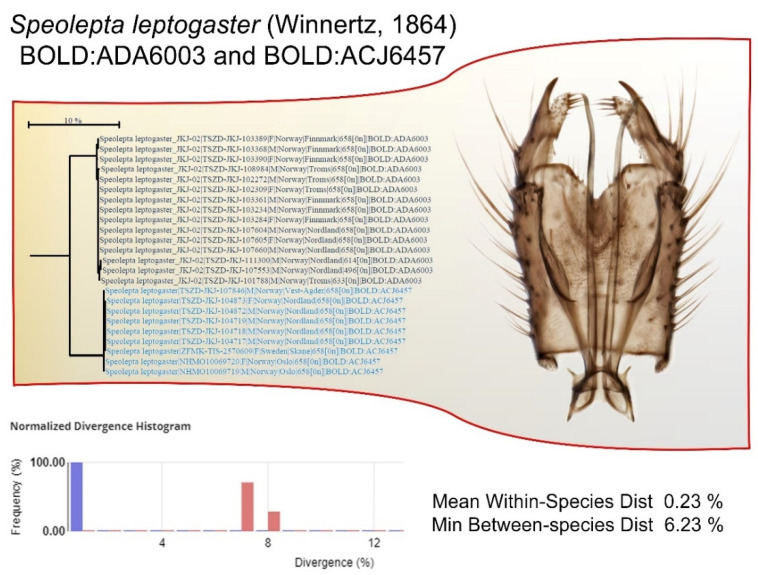
The cave-dwelling mycetophilid species *Speolepta leptogaster* (Winnertz, 1864) is an example of BIN splitting without any discernible morphological segregation. Two distinct BINs are separated with a minimum between-species distance of 6.23%, suggesting that two cryptic species are involved.

In some cases, no morphological differentiation at all is found even when the genetic differentiation gives rise to deep BIN splits. The cave-dwelling mycetophilid species *Speolepta leptogaster* (Winnertz, 1864) is a good example of this (Figure 8). The species is split into two BINs with a minimum between-species distance of 6.23% and a mean within-species distance of 0.23%. The BIN BOLD:ADA6003 displays a northern distribution, in Norway, ranging down to Nordland county. The BIN BOLD:ACJ6457 displays a southern, continental distribution with its northern range reaching Nordland county in Norway.

Despite this distinct genetic segregation, no differentiation has yet been found to separate them morphologically. *Speolepta leptogaster* (Winnertz, 1864), thus, serves as a possible case of a real cryptic species split (in the meaning that cryptic species cannot be morphologically separated by humans). It is interesting to note that this occurs for a cave-dwelling species that might have quite isolated populations, although representatives of both BINs were found at the same epigean locality in Nordland. In the revision of the genus *Speolepta* Edwards, 1925, Ševčík et al. [82] discuss the apparent great dispersal potential for the adults despite its species being obligate cave-dwellers at the larval stage. A haplotype network study [83] based on immature stages from Germany also concluded a good and active dispersal ability of *Speolepta leptogaster*, but as this study sequenced the third position (COI-3P) it cannot be directly compared with standard DNA barcodes based on the fifth position (COI-5P). This speciation case is not yet concluded, but the author hesitates to describe new species without being able to distinguish them morphologically. Rather, in such cases, the BIN registry may serve as additional information about the population-level segregation of a species with large genetic differentiation.

Real cryptic species will, by their nature, have an elusive status since they are only accessible to those with the means and funding to sequence or barcode their samples in a specific manner. Hence, such cryptic “barcode species” may be better characterized only by their genetic characteristics without being given new Linnean names, a proposition held, e.g., by Ahrens et al. [84]. In this context, it should be noted that for herbivorous, gall-forming Cecidomyiinae, a different practice of species diagnosis is sometimes used. For example, since the males of some *Asphondylia* Loew, 1850, are considered indistinguishable, alternative diagnostic characters are searched for only in the females, larvae, pupae, gall morphology, ecology, or genetics [60,63]. How this is affecting the species concept across different taxa of Sciaroidea is an open question.

### 2.4. Secondary DNA Barcoding Outside of BOLD

While the BOLD database online may have almost a monopoly on DNA barcoding of insects, a lot of COI sequences that can be and are used for barcoding are produced by researchers independently and outside of BOLD. These are mainly used for molecular phylogenetic studies [1,37,85,86,87,88,89,90,91,92,93,94,95] and deposited in GenBank, but also include taxonomic descriptions and revisionary works [43,64,67]. BOLD and GenBank have an exchange agreement such that all the COI sequences in GenBank are harvested into BOLD. A search on BOLD for Sciaroidea sequences mined from GenBank resulted in 5463 sequences of which 5288 were COI sequences (Supplementary Materials). These make up 1084 BINs and are reported on BOLD to represent 1083 species. The BIN list of named COI sequences, however, is reduced to 890 species names (including interim names). Hence, these names make up another substantial bulk (31%) of all the named BINs on BOLD, and they consist largely of high-quality identifications often representing species belonging to rarely studied taxa from all over the world.

### 2.5. Alternatives to BOLD

Sciaroidea flies have demonstrated a surprisingly good overall match between Linnean names based on morphology and the automated Refined Single Linkage (RESL) method used on BOLD to assign BINs. Sevcik et al. [39] compared the utility of the standard COI gene region with three other regions (COII, CytB, and ITS2) for European species of the mycetophilid genus *Docosia* Winnertz, 1864. They found CytB to be the best barcoding marker, closely followed by COI while ITS2 performed the worst, a result also shared by Jürgenstein et al. [37] and Kurina et al. [36]. A major reason for the good match to the COI mitochondrial evolutionary clock may be sourced in the relatively young age and rapid radiation of many recent fungus gnats, as demonstrated for the mycetophilid tribe Exechiini [93]. Far from all insect taxa demonstrate the same success on BOLD [96]. Thus, for Sciaroidea, the BIN registry has been adopted by the author in favor of alternative methods such as Automatic Barcode Gap Discovery (ABGD) [97] and Poisson Tree Processes (PTP) [98]. Hartop et al. [23] argue for combining several methods into a Large-scale Integrative Taxonomy (LIT) workflow, but then only applying different methods for those taxa that show ambiguous results after the first round of cost-efficient barcoding. This is indeed a promising way to approach the problematic cases with BIN vs. morphology conflicts, but ultimately, for the author, the match back to morphology will always be decisive to delimit species in a practical manner.

New methods and increasing access to genomic data will undoubtedly soon change and develop the barcoding enterprise beyond today’s BOLD. MinION, an affordable, portable sequencer, is now ready for low-cost, large-scale biodiversity discovery across the globe [99], and next-generation sequencing tools are being applied [100]. Still, these efficient new tools will only be as good as the reference libraries they depend upon, and a great advantage of BOLD is that it is increasingly used worldwide to assemble in one infrastructure the largest cross-comparable barcode database ever.

### 2.6. There Is Hope for You Yet, Taxonomy

Cotterill and Foissner [101] make a strong philosophical case for how rigorous biodiversity inventories and taxonomy underpin scientific knowledge and challenge us to survey biodiversity representatively by detailing the natural history of species. Using BINs and dark taxa to evaluate biodiversity and conduct protection management is a shortcut that in their words results in an “*incomplete ‘Brochure of Life’* [that] *cannot match the scientific integrity of the ‘Encyclopedia of Life*’’ (abstract in [101]). Focusing on BINs and dark taxa is a charismatic and understandable shortcut that has an inevitable impact on the modern academic environment by virtue of the advanced technology that can greatly speed up the rate of discovery. However, it comes with a backlash to both traditional and integrative taxonomy. Once the magnitude of the fauna is disclosed by such BINs-only surveys it may become even harder to obtain a project to fully describe or review the fauna funded. Who is going to invest in morphological revisions of, e.g., the gall midges of Krüger National Park in South Africa after it is disclosed by D’Souza et al. [19] that the area has 2162 BINs of dark taxa? One should really ask how a project to morphologically review, for instance, some 100 species of Sciaroidea can compete with such BINs-only approaches. A different example, outside the Sciaroidea, that has recently raised discussions, is the minimalist revision of Costa Rican brachionid parasitoid wasps based on barcodes alone by Sharkey et al. [102] (see discussion by Ahrens et al. [84] and Meier et al. [103]). Sharkey et al. [102] used barcodes and BINs not only to estimate the magnitude of the fauna but introduced them as new tools for a high-speed minimalistic taxonomy workflow to lead the way, absent resources to conduct a full integrative revision. In doing so, they succeeded in speeding up the naming process but left behind a messy and incomplete legacy that requires a full integrative revision in order to move forward.

Given the large amount of Sciaroidea barcodes already present on BOLD, an opportunity exists now to relocate funds and efforts from the initial, blind barcode scanning towards full integrative studies in order to build the reference library for the 95% dark taxa already sequenced. Provided that high-quality vouchers are being deposited in publicly available collections for the majority of the BINs, a new field of “integrative barcode revisions” can be encouraged and funded. For Sciaroidea as a whole, such a task has the potential to more than triple the known world fauna without much additional sampling. Examples of such “integrative barcode revisions” on a small scale include the recent revision of the *Exechia parva* group by Lindemann et al. [45] and the description of the genus *Coelosynapha* by Kjærandsen et al. [44]. In both of these studies, the BOLD archive was used extensively to borrow barcoded vouchers and, in this way, included species from North America in the revisionary work. A problem arose that the quality of the vouchers in the BOLD archive, which are stored in ethanol and often contain fragmented specimens, was not always of the standard needed for long-term preservation of type materials. In most cases, however, types from each BIN representing new species to science could be selected, dried, and pinned. For tiny species, like most gall midges, a poor voucher quality of specimens representing the BOLD archive is likely to pose a major problem and additional sampling will be required to select type specimens that are associated back to those originally barcoded.

As demonstrated with the Nordic initiative, high-quality reference libraries of pinned or slide-mounted specimens can indeed be achieved after a rigorous post-sequence revision that can enable unequivocal BIN identification of some 90–95% of the species. The remaining 5–10% will need even more detailed population-level studies to uncover haplotype networks of complex species relations, as demonstrated for birds [104]. As such, results on DNA barcoding of Sciaroidea from the Nordic region are in line with great success rates reported after a rigorous revision for several other, better-studied taxa, such as gracillariid moths [105,106].

## 3. Conclusions

After two centuries of morphological exploration of Sciaroidea flies, DNA barcoding has quickly integrated with traditional taxonomy in a boosted phase of new frontier exploration of Sciaroidea diversity worldwide. More than 1.2 million sequences and 56,000 BINs of Sciaroidea flies now assembled on BOLD have the potential to more than triple the known fauna of Sciaroidea, but as of today, 95% of the BINs remain unnamed, dark taxa only figuring in publications as statistics representing proxies for species diversity within a family. Integrative taxonomic studies of Sciaroidea taxa in descriptive and revisionary works have helped to build a rigorous reference library on BOLD but so far only make up a small fraction of the entire database.

The dark taxa are strongly biased towards the megadiverse and less-studied families Cecidomyiidae and Sciaridae, while for Mycetophilidae, Keroplatidae, and the remaining smaller families a considerably larger proportion of the BINs are already assigned to named species. The latter is thanks to Nordic initiatives in Norway and Finland, where the local nodes of iBOL, NorBOL, and FinBOL, over the last decade, have engaged trained taxonomists to fully barcode their native insect fauna. Additionally, mined DNA barcodes from GenBank into BOLD constitute a considerable source of named taxa.

In the rigorous process of building the Nordic reference library of Sciaroidea, patterns of mismatches between BIN assignments and traditional morphological identification can be grouped into three major categories: (1) An error rate of some 5% in morphological species identification pre-sequence detected post-sequence that, such as contaminations and lab mix-ups, can and should be corrected; (2) numerous semi-cryptic species being newly discovered to science by integrative iterations back and forth between ID trees based on DNA barcodes and morphological studies; and (3) numerous real and confirmed mismatches between morphology and BINs, highlighting both cases of BIN sharing of morphologically distinctly different species and BIN splitting without any apparent morphological or ecological differentiation. These can partly be fine-tuned with manual BIN calibrations. Barcoded species from the Nordic region now display a mean distance to the nearest neighbor of 6.97% while the mean intra-specific distance is 0.81%. This indicates on average some 45 COI base pairs being different between closely related species of Sciaroidea.

The potential of DNA barcoding and its BIN registry on BOLD to represent the natural history of Sciaroidea species will never be fully reached without a wholehearted and better founded and funded engagement of trained taxonomists to continually build, curate, and revise the associated reference libraries and describe new species from the accumulated black box of dark taxa. The work required to achieve high-quality reference libraries for species rich and under-studied taxa, such as the Sciaroidea, can be likened to family-level taxonomic revisions. The opportunity for such “integrative barcode revisions” should be encouraged. Only when the BINs are named—properly and partly manually calibrated by a rigorously quality-checked reference library—the great potentials of both classical taxonomic barcoding, metabarcoding, and eDNA ecology can be realized for Sciaroidea.

## Figures and Tables

**Table 1:** Statistics of DNA barcoded Diptera belonging to superfamily Sciaroidea extracted familywise from BOLD as of 14 October 2021. DS-FINPRO is a published dataset on BOLD serving as a Finnish reference library presented by Roslin et al. [26]. Norwegian published species are extracted from Elven and Søli [27] with additions on the Mycetophilidae by Kjærandsen and Søli [24] and some new data extracted from GBIF. The Swedish estimate is taken from Ronquist et al. [4]. The Nordic estimate is original, including unpublished data in progress of being published, with an estimate for the Cecidomyiidae kindly mediated by M. Jaschhof (*pers. com.*). Abbreviations: BINs = Barcode Index Numbers, BOLD = Barcode of Life online database BoldSystems, ID species = morphologically identified species, SPM = specimens, SPP = species.

Taxon	BOLD, World Totals (14 October 2021)							
	Specimens	% BOLD	BINs	% BOLD	ID Species	% ID Species	SPM/BINs	BINs/SPP
**Sciaroidea**	**1,224,877**	**9.2%**	**56,648**	**9.5%**	**2843**	**5.0%**	**22**	**20**
Cecidomyiidae	661,414	5.0%	43,763	7.3%	594	1.4%	15	74
Sciaridae	479,666	3.6%	9077	1.5%	755	8.3%	53	12
Mycetophilidae	73,622	0.6%	3267	0.5%	1301	40%	23	3
Keroplatidae	8500	0.1%	435	0.07%	134	31%	20	3
Bolitophilidae	515	0.004%	37	0.006%	35	95%	14	1
Ditomyiidae	823	0.006%	40	0.007%	6	15%	21	7
Diadocidiidae	329	0.002%	26	0.004%	15	58%	13	2
*Incertae sedis*	8	0.0001%	3	0.001%	3	100%	3	1
**Taxon**	**Nordic barcoding, including private data**							
	Specimens	% BOLD	BINs	% BOLD	ID species	% BOLD	SPM/BINs	BINs/SPP
**Sciaroidea**	**14,908**	**1.22%**	**2191**	**3.9%**	**1557**	**55%**	**7**	**1.4**
Cecidomyiidae	2560	0.39%	614	1.4%	81	14%	4	8
Sciaridae	4972	1.04%	601	6.6%	414	55%	8	1.5
Mycetophilidae	6685	9.08%	877	27%	964	74%	8	0.9
Keroplatidae	391	4.60%	54	12%	55	41%	7	1.0
Bolitophilidae	217	42%	36	97%	34	97%	6	1.1
Ditomyiidae	13	1.58%	2	5.0%	2	33%	7	1.0
Diadocidiidae	66	20%	6	23%	6	40%	11	1.0
*Incertae sedis*	4	50%	1	33%	1	33%	4	1.0
**Taxon**	**Nordic species estimates as proportion of Nordic BINs**							
	DS-FINPRO ref. library	BINs/DS-FINPRO	Norwegian published species	BINs/Norwegian species	Swedish species estimate	BINs/Swedish estimate	Nordic species estimate	BINs/Nordic estimate
**Sciaroidea**	**440**	**4.98**	**1289**	**1.70**	**2720**	**0.81**	**3727**	**0.59**
Cecidomyiidae	16	38.38	245	2.51	1250	0.49	1800	0.34
Sciaridae	81	7.42	143	4.20	470	1.28	750	0.80
Mycetophilidae	305	2.88	835	1.05	890	0.99	1050	0.84
Keroplatidae	33	1.64	38	1.42	60	0.90	75	0.72
Bolitophilidae	2	18.00	21	1.71	38	0.95	40	0.90
Ditomyiidae	0	-	2	1.00	3	0.67	3	0.67
Diadocidiidae	3	2.00	4	1.50	8	0.75	8	0.75
*Incertae sedis*	0	-	1	1.00	1	1.00	1	1.00

## Data Availability

See the Supplementary Materials.

## Supplementary Materials

Two datasets on BOLD are made publicly available with this publication. The first contains a dataset of Sciaroidea taxa submitted by the author to BOLD, chiefly from the Nordic region but also containing some sequences from other areas across the Holarctic region. This is available in the public dataset DS-NORSC (https://doi.org/10.5883/DS-NORSC) on BOLD. The second contains a dataset of Sciaroidea taxa mined from GenBank into BOLD. This is available in the public dataset DS-SCIBLAST (https://doi.org/10.5883/DS-SCIBLAST) on BOLD.
